# Genome-Wide Characterization and Expression Analysis of *CYP450* Genes in *Chlamydomonas reinhardtii* P.A. Dang.

**DOI:** 10.3390/biology15010077

**Published:** 2025-12-31

**Authors:** Runlong Zhou, Xinyu Zou, Fengjie Sun, Yujie Kong, Xiaodong Wang, Yuyong Wu, Chengsong Zhang, Zhengquan Gao

**Affiliations:** 1 School of Pharmacy, Binzhou Medical University, Yantai 264003, China; xycrl21@163.com (R.Z.); 15698067037@163.com (X.Z.); 19508662291@163.com (Y.K.); plamanwxd@outlook.com (X.W.); wuyuyong_820@126.com (Y.W.); 21410010898@stumail.sdut.edu.cn (C.Z.); 2 Department of Biological Sciences, School of Science and Technology, Georgia Gwinnett College, Lawrenceville, GA 30043, USA; fsun@ggc.edu

**Keywords:** CYP450, gene family, *Chlamydomonas reinhardtii*, expression analysis

## Abstract

Cytochrome P450 (CYP450) monooxygenases are a class of enzymes containing conserved heme-binding functional domain. They contribute to a wide range of biosynthetic processes, serving a pivotal function in plant resistance to abiotic stress. To date, little is known about the CYP450s of *Chlamydomonas reinhardtii*. In our study, a total of 37 *crP450* genes were identified from *C. reinhardtii* based on domain and sequence alignment, unevenly distributed on 12 chromosomes with 4 pairs of tandem replications shared among family members. Most of these genes contained 10 or more introns and encoded CYP450 proteins with an average of 593 amino acids and 3–9 conserved motifs. CYP450 enzymes were mainly distributed in the chloroplasts, cytoplasms, mitochondria, and cytoplasmic membranes. There were numerous light, jasmonic acid, abscisic acid, and salicylic acid response elements located in the upstream of gene coding sequences, suggesting that these genes could be modulated by plant hormones. Transcriptome analysis uncovered distinct expression patterns of *crP450* genes under various stress conditions, with the 37 *crP450* genes grouped into 9 clusters. In summary, this study presented a genome-wide characterization of *CYP450* genes in *C. reinhardtii*, providing a strong foundation for further exploration into their biological functions.

## Simple Summary

Enzymes are essential for life processes in all organisms. One specific family of enzymes, known as Cytochrome P450, plays a critical role in helping plants and animals synthesize essential compounds and defend against environmental stress. However, scientists currently possess limited knowledge about these enzymes in *Chlamydomonas reinhardtii*, a single-celled green alga widely used as a model for biological research. This study aimed to identify all the Cytochrome P450 genes in this alga and investigate how they function under stress. We identified 37 specific genes and mapped their physical characteristics and locations within the cell. By exposing the algae to harsh conditions—including high salt, intense light, low temperature, and nutrient shortages—we discovered that these genes become highly active to help the algae survive. Specifically, we found that distinct groups of genes are triggered by different threats, such as iron deficiency or salt stress. These findings are valuable to society because they reveal the genetic mechanisms algae use to withstand environmental challenges. These insights provide a crucial foundation for future strategies to improve algal stress resilience, facilitating their broader application in sustainable biotechnology.

## 1. Introduction

Cytochrome P450 (CYP450) monooxygenases are encoded by a supergene family and widely distributed across all forms of life, including animals, plants, protists, fungi, bacteria, archaea, and even viruses [1]. CYP450 enzymes are involved in many metabolic processes, e.g., drug metabolism, hormone synthesis, and fatty acid metabolism [2]. They catalyze the insertion of the oxygen atom into hydrophobic molecules, making them more reactive or hydrophilic. The name “CYP450” is derived from their ability to bind with the reducing carbon monoxide and the maximum absorption peak occurring at 450 nm [3]. To date, more than 300,000 *CYP450* genes have been found due to the explosive growth in genome sequencing data. Numerous microbial CYP450 catalytic systems require both oxidizing and reducing partners to function. In the form of separate protein or CYP450 fusion domain, two electrons are transmitted from NAD(P)H to the Hemorrhin Iron Reacting Center and used for O_2_ activation [4,5]. The conserved heme-binding domain is characterized as the key determinant for classifying enzymes into the CYP450 family. Four α-spiral structures (D, L, I, and E) form a conserved core of CYP450, which contributes to a wide range of biosynthetic processes, such as pyrine biosynthesis pathway, fatty acid metabolism, and pigment synthesis [6,7]. A group of five CYP450s (i.e., CYP72A219, CYP72A15, CYP97B2, CYP71A1, and CYP86A8) were identified as candidates involved in crocin biosynthesis in *Crocus sativus* L. [8]. Therefore, the identification and characterization of *CYP450* genes play important roles in further exploring terpene biosynthesis pathway, fatty acid metabolism, and pathways of pigment synthesis. In addition, in *Arabidopsis thaliana*, CYP450s also participate in the jasmonic acid metabolic pathway, thereby influencing the plant response to stress conditions [9].

Algae constitute a highly varied assemblage of aquatic organisms, spanning from small, unicellular cyanobacteria to large, meter-scale giant seaweeds [10,11]. Most algae are photosynthetic organisms, accounting for about 50% of the total photosynthesis on Earth [12]. Algae have demonstrated significant potential as versatile biotechnological platforms, spanning from the generation of biofuels and feedstocks to the accumulation of high-value compounds [13]. This potential is largely attributable to their facile cultivation, fast growth cycle, phenotypic plasticity, and pronounced ability to amass molecules relevant to biofuels (e.g., lipids and starch) or commercially important secondary metabolites (e.g., β-carotenoids and astaxanthin), solidifying their status as a leading model system in research [14]. For example, *Chlamydomonas reinhardtii* has long served as a model organism for studying photosynthesis and flagellar motility [15]. However, recent advances have positioned this model organism as a premier platform for biotechnological applications, particularly in bioremediation [16] (e.g., heavy metal and wastewater treatment) and the sustainable production of bioproducts such as lipids, recombinant proteins, and pigments. The availability of advanced genetic toolkits, including CRISPR-Cas9 editing [17] and the comprehensive library of indexed tagged mutants available at the Chlamydomonas Resource Center (CLiP) [18], further supports its utility in metabolic engineering. In particular, insertional mutagenesis has emerged as a critical strategy not only for functional genomics but also for unlocking novel metabolic pathways. High-throughput resources, such as the indexed mutant library from the CLiP, allow for genome-wide saturation mutagenesis to identify essential regulatory nodes. Recent studies demonstrate that this approach can effectively ‘open’ new metabolic routes by disrupting negative regulators or competing pathways, thereby alleviating feedback inhibition and redirecting carbon flux toward the overproduction of high-value metabolites like lipids and carotenoids. These resources make *C. reinhardtii* an ideal organism for elucidating gene function and developing robust biotechnological applications [19]. Meanwhile, *C. reinhardtii* has emerged as the primary reference organism for investigating algal–microbial mutualism and synthetic ecology [20]. Recent studies have utilized this model to decipher the complex metabolic exchanges, such as vitamin and carbon trading as well as signaling networks within the phycosphere, offering new insights into how microalgae orchestrate interactions with beneficial bacterial communities [21]. In recent years, *C. reinhardtii* has been increasingly studied and recognized as a model system for the study of key metabolic pathways, including photosynthesis, primary metabolism, intercellular organelle communication, and stress response [22], due to its three fully sequenced genomes and its capability for genetic transformation [23]. In addition to its economic significance and non-toxic properties, this alga possesses a post-translational modification system that preserves the functional activity of recombinant proteins, matching their natural counterparts. Additionally, it can utilize acetate as the sole carbon source for non-photosynthetic growth, making it an ideal platform for applications in molecular biology and other fields [24]. For example, the chloroplasts of *C. reinhardtii* have been transformed into an economical and readily scalable platform for generating therapeutic recombinant proteins [25,26,27]. This alga is also considered a potential biofuel-producing microorganism due to its production of large amounts of biomass through photosynthesis and accumulation of important biofuel precursors. Specifically, starch granules accumulate primarily within the chloroplast [28], while neutral lipids (triacylglycerols) are stored in cytosolic lipid droplets [29], particularly under nitrogen-deficient conditions. Furthermore, recent trends in biotechnology have shifted toward the use of *C. reinhardtii* in algal-bacterial consortia rather than strict monocultures. These symbiotic systems utilize metabolic exchange to significantly enhance biomass stability and lipid productivity [30] while simultaneously improving the efficiency of pollutant detoxification in bioremediation applications compared to axenic cultures [31]. It can be used as a bioindicator for environmental pollution because it is highly sensitive to environmental pollutants. For example, *C. reinhardtii* has been used for wastewater purification as researchers can assess the level and impact of pollution in the environment by observing the growth and metabolism of *C. reinhardtii* under different pollutant concentrations [32].

While genome-wide surveys of CYP450 genes have been reported in various algal taxa, the diversity of these genes varies widely among taxa. For example, the chlorophyte *Volvox carteri* has been reported to encode approximately 19 CYP genes based on comparative genomic data in plant CYP surveys [33], although the exact number depends on current annotations. Current knowledge is largely fragmented, focusing on isolated enzymes rather than the family’s broad evolutionary and functional landscape. This study addresses this gap by providing the first comprehensive genome-wide identification and expression profiling of the *C. reinhardtii* CYP450 family. By clarifying the phylogenetics, subcellular localization, and stress-responsive patterns of these genes, we aim to provide a foundational roadmap for future functional studies and metabolic engineering efforts in this key model organism. In this study, the *CYP450* genes in *C. reinhardtii* were identified using bioinformatics methods, with their conserved loci revealed using comparative sequence analysis. The cellular distributions of CYP450s were clarified, and their molecular and physical properties were further characterized. Finally, the expression patterns of *CYP450* genes under different environmental conditions were evaluated using transcriptome analysis and quantitative real-time PCR (qRT-PCR). The goals of this study were to identify and characterize the *CYP450* genes in *C. reinhardtii* and to provide a strong reference for further exploration of the *CYP450* gene family in *C. reinhardtii*.

## 2. Materials and Methods

### 2.1. Microalgal Strain and Culture Conditions

*Chlamydomonas reinhardtii* strain FACHB-265 (a wild-type strain with intact cell walls) was obtained from Freshwater Algae Culture Collection at the Institute of Hydrobiology (FACHB), National Aquatic Biological Resource Center (Wuhan, China), and cultured in TAP medium (Table S1) at 25 °C under light (100 µmol photons m^−2^ s^−1^). The morphological features of the *C. reinhardtii* cells and the culture conditions were shown in Figure S2. To analyze gene expression patterns, log-phase cells were subjected to eight experimental conditions for 7 d. Cultures were inoculated at an initial density of OD_750_ = 0.10 and subjected to eight experimental conditions for 7 d. This duration was specifically selected to evaluate the long-term transcriptional acclimation of the cells and the regulation of secondary metabolism (lipid and pigment accumulation) characteristics of the late-log or stationary phases under chronic stress, including a normal control, five single-factor stresses (high light 400 µmol photons m^−2^ s^−1^, high salt 150 mM NaCl, low temperature 16 °C, iron deficiency, and phosphorus deficiency), and two combined stresses (iron/phosphorus deficiency with high salt 150 mM NaCl; high light with high salt 150 mM NaCl).

### 2.2. Identification of the CYP450 Gene Family Members in Chlamydomonas reinhardtii

The genomic sequences and annotation files of *C. reinhardtii* (version v5.5) were downloaded from the Ensembl Plants database (Release 57) (https://plants.ensembl.org, accessed on 10 September 2023). The Hidden Markov Model (HMM) profile of the CYP450 domain (PF00067) was retrieved from the Pfam database (v35.0) (http://pfam.xfam.org/, accessed on 15 October 2023) [34]. HMMER 3.3 [31] was used to align *C. reinhardtii* CYP450 protein sequences (E-value < 10^−10^). To further verify the resulting CYP protein sequences, Blast2GO was used to annotate the predicted CYP450 sequences using SwissProt database (E-value < 10^−3^) to ensure broad functional coverage across species, and BLASTP 2.16.0 was performed to obtain the alignment (E-value < 10^−3^) using *Arabidopsis* CYP450 protein sequence (TAIR10). *Arabidopsis* was selected as the reference species due to its standard nomenclature for plant CYP450 clans, allowing for accurate phylogenetic classification of the algal candidates. The identified CYP450 protein sequences of *C. reinhardtii* were uploaded to the online tool CCD (https://www.ncbi.nlm.nih.gov/Structure/cdd/cdd.shtml, accessed on 10 Januray2024) [35] for domain prediction (E-value < 10^−10^), and the sequences with incomplete domain were removed. Tophat 2 [36] was used to map the transcriptome data to the genome of *C. reinhardtii*, and sequences with annotation errors were removed. The isoelectric point (pI) and molecular mass (MW) of the CYP450 sequences were calculated using the online tool Compute PI/MW (https://web.expasy.org/compute_pi/, accessed on 25 May 2024) [37]. The online tool WoLF PSORT (https://wolfpsort.hgc.jp, accessed on 21 August 2024) [38] was used for subcellular location prediction, and the online tool SignalP 5.0 (https://services.healthtech.dtu.dk/services/SignalP-5.0/, accessed on 15 December 2024) [39] was used for signal peptide prediction.

### 2.3. Sequence Analysis of the crP450 Genes in Chlamydomonas reinhardtii

The exons and introns of *crP450* genes of *C. reinhardtii* were evaluated by TBtools 2.0 and online tool GSDs 2.0 (https://gsds.gao-lab.org/, accessed on 7 March 2025) [40]. The online tool MEME 5.5.9 (https://meme-suite.org/meme/tools/meme, accessed on 17 April 2025) [41] was used to identify conserved motifs in the CYP450 protein sequence of *C. reinhardtii*. The consensus amino acid sequence and the conservation level of specific domains were visualized using the WebLogo 3 server (https://weblogo.threeplusone.com/, accessed on 31 July 2025) [42] based on the crP450 proteins.

### 2.4. Phylogenetic Analysis of CYP450 Sequences in Chlamydomonas reinhardtii

The multisequence alignment was performed using MUSCLE, and the trimming of the aligned protein sequences was completed using trimal [43]. A neighbor-joining tree was constructed using MEGA 10.0 [44], with bootstrap support obtained using 1000 replicates, gap/missing data treated with partial deletion, and site coverage cutoff set to 80.

### 2.5. Chromosomal Localization and Gene Duplication of the crP450 Genes in Chlamydomonas reinhardtii

Chromosomal distributions of *crP450* genes were revealed using *C. reinhardtii* genome files and genome annotation files. Gene duplication events in *CYP450* genes were determined using MCScan 10.0 [45].

### 2.6. Analysis of Cis-Acting Elements in the Promoter Regions of crP450 Genes in Chlamydomonas reinhardtii

The upstream sequence region (2 kb) of the coding sequence of the *crP450* genes in *C. reinhardtii* was analyzed using the online tool PlantCARE (https://bioinformatics.psb.ugent.be/webtools/plantcare/html/, accessed on 31 July 2025) [46] to identify the *cis*-acting elements in the promoter regions of the *CYP450* genes. Additionally, specific searches were conducted for nitrogen-responsive GATA-box motifs (binding sites for the NIT2 transcription factor) to evaluate potential regulation by nitrogen status.

### 2.7. Functional Analysis of the crP450 Genes in Chlamydomonas reinhardtii

The functional annotation of the *crP450* genes was performed using KEGG (https://www.kegg.jp, accessed on 7 August 2025) and Uniprot (https://www.uniprot.org, accessed on 7 August 2025) databases.

### 2.8. RNA Sequencing Data Analysis

Transcriptome data of *C. reinhardtii* were downloaded from the NCBI SRA database (https://www.ncbi.nlm.nih.gov/sra, accessed on 16 September 2024) under six different conditions, including short high-light treatment (SRR10737780), iron deficiency treatment (SRR10290597), phosphorus deficiency treatment (SRR10290598), high-salt treatment (SRR10290596), low-temperature (16 °C) treatment (SRR10290595), and normal treatment (SRR10290600). Prior to alignment, raw reads were subjected to rigorous quality control using FastQC. Low-quality reads (Phred score < 30), poly-N stretches, and adapter sequences were removed using Trimmomatic to generate clean reads. High-quality reads were then mapped onto the *C. reinhardtii* reference genome (v5.5, Ensembl Plants Release 57) using TopHat2 [29], allowing for a maximum of two mismatches. Samples with a mapping rate of >75% were selected for further analysis. Gene expression levels were calculated based on Fragments Per Kilobase of transcript per Million mapped reads (FPKM) using Tophat2. FPKM values were calculated by Cufflinks [47] and normalized to generate the heatmaps of *CYP450* gene expression levels.

### 2.9. Quantitative Real-Time PCR Analysis of crP450 Gene Expression

To validate the expression patterns revealed by the transcriptome analysis, *C. reinhardtii* samples were collected from seven experimental groups: five single-factor stress conditions (high light, iron deficiency, phosphorus deficiency, high salt, and low temperature) and two combined stress conditions (iron/phosphorus deficiency with high salt; high light and high salt). Samples cultured under normal conditions served as the control. Samples of *C. reinhardtii* were ground in liquid nitrogen [25] and used to extract total RNA using TRIZOL reagent [48]. The first strand of cDNA was prepared using SuperScript™ III Reverse Transcriptase Kit (Vazyme, Nanjing, China). qRT-PCR of *crP450* genes was performed using Light Cycler 9600 system (Roche, Basel, Switzerland) and 2 × Universal SYBR QPCR Mix (Rui Biotech, Beijing, China). The qPCR amplification procedure began with an initial denaturation at 95 °C for 30 s, followed by 40 cycles of denaturation at 95 °C for 10 s and primer annealing at 60 °C for 30 s. The *C. reinhardtii CBLP* gene was used as the internal reference gene for normalization. The stability of *CBLP* expression under the tested stress conditions (high light, salt, and nutrient deficiency) was validated experimentally [49], with threshold cycle (Ct) variations maintained within ±0.5 cycle across all treatment groups.

### 2.10. Statistical Analysis

The expression levels of *crP450* genes were calculated using 2^−ΔΔCT^ method based on three biological replicates of qRT-PCR experiments, with three technical replicates per biological replicate performed. Statistical differences were determined using multiple *t*-tests with the Holm–Sidak method to correct for multiple comparisons in GraphPad Prism version 10.0.0. Differences were considered statistically significant based on *p* < 0.05.

## 3. Results

### 3.1. Identification of CYP450 Genes in Chlamydomonas reinhardtii

A total of 44 members were initially identified by HMMER. Then, 2 sequences were removed based on BLAST analysis, 2 sequences were deleted based on CCD analysis and transcriptome validation, and 3 redundant sequences were removed as potential false positives or duplicates to ensure the accuracy of family identification. Finally, a total of 37 non-redundant and high-confidence sequences of *CYP450* gene family in *C. reinhardtii* were obtained and designated as *crP450-1* to *crP450-37* (Table 1). The CYP450 proteins ranged from 381 to 1029 amino acids in length (average 593 amino acids and median 586 amino acids), with molecular mass (Da) ranging from 41,742.8 to 107,301.3 and pI values ranging from 5.6 to 9.6. *CYP450* genes with an intron count ≤ 0 accounted for 38% of the total number of genes, genes with an intron count > 10 and ≤15 accounted for over 57% of the total number of genes, and genes with the intron count > 15 accounted for 5% of all *crP450* genes. The subcellular localization of majority of crP450 (20) was predicted to be in chloroplast, followed by mitochondria (5), cytoplasm (4) and plasma membrane (4), endoplasmic reticulum (2), and one each for the extracellular and nuclear membrane. A total of four sequences, including crP450-1, crP450-9, crP450-20, and crP450-33, were predicted for the presence of signaling peptides. Specifically, SignalP 5.0 predicted cleavage sites between amino acids 23–24 for crP450-1, 37–38 for crP450-9, 25–26 for crP450-20, and 28–29 for crP450-33, consistent with their translocation across cellular membranes.

### 3.2. Phylogenetic and Sequence Analyses of crP450 Genes in Chlamydomonas reinhardtii

**Figure 1:**
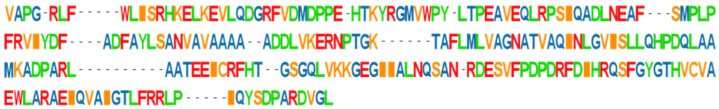
Consensus amino acid sequence of the CYP450 gene family in *Chlamydomonas reinhardtii*, representing the most frequent amino acid residues derived from the multiple sequence alignment of all 37 identified crP450 proteins. Colored letters indicate the chemical properties of the amino acids based on the ClustalX scheme, i.e., charged residues (red), polar uncharged residues (green), hydrophobic residues (orange), and aromatic residues (blue). The symbol “-“ represents gaps introduced to align the diverse members of the family. The Sequence Logo illustrating the conservation of these domains is provided in Figure S1.

**Figure 2:**
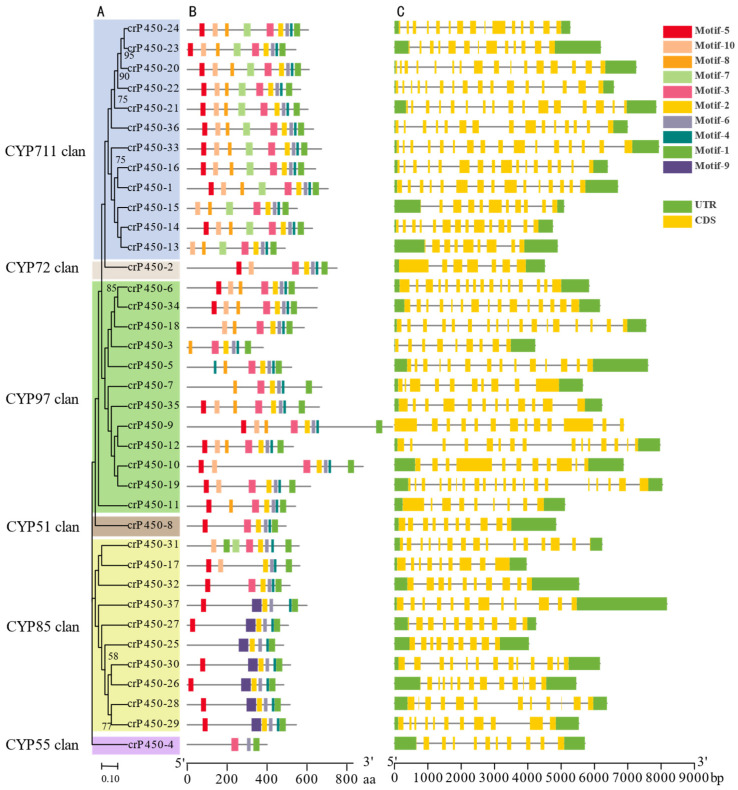
Phylogenetic tree of crP450 protein sequences (**A**), conserved crP450 protein motifs (**B**), and *crP450* gene structure (**C**) of *Chlamydomonas reinhardtii*. The rulers represent the number of amino acids and the nucleotide length (base pairs) in (**B**) and (**C**), respectively.

The multiple sequence comparison of the 37 crP450 protein sequences was performed to reveal the conserved regions of protein sequences of *C. reinhardtii* CYP450 family (Figure 1). The phylogenetic tree was reconstructed based on *C. reinhardtii* CYP450 protein sequences, demonstrating the division of CYP450 proteins into six clans (Figure 2). MEME was used to identify a total of 10 types of conserved motifs in the *CYP450* gene family of *C. reinhardtii*, showing that the number and distribution of all 10 types of motifs were largely comparable, while the number of motifs differed between different types of motifs (Table 2). GSDS was used to analyze the distribution of *CYP450* exons, showing that most of the *CYP450* genes contained numerous short exons, indicating the variations among *CYP450* gene family members.

### 3.3. Chromosomal Distribution of crP450 Genes in Chlamydomonas reinhardtii

**Figure 3:**
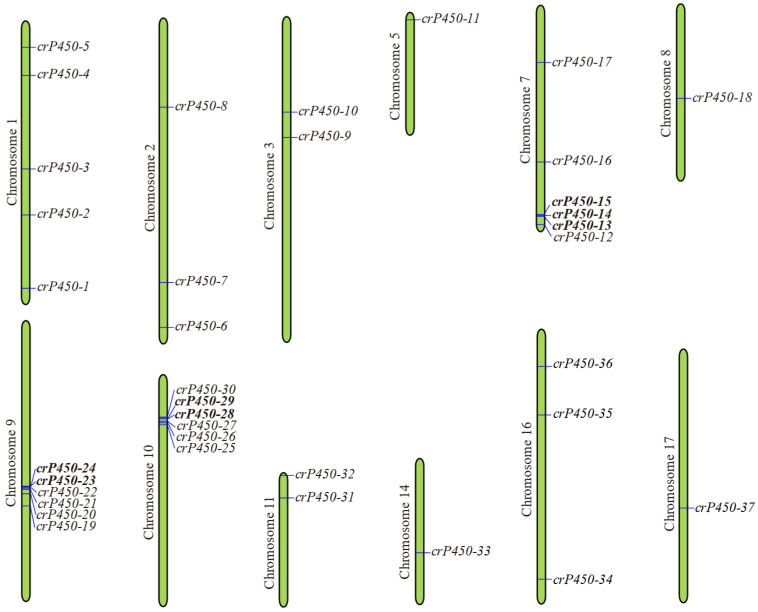
Chromosomal locations of *crP450* genes in *Chlamydomonas reinhardtii*. Two genes in tandem repeat sequences are indicated in bold.

The chromosomal distribution of *crP450* genes was heterogeneous, spanning a total of 12 chromosomes (Figure 3). The highest number of *crP450* genes was localized on chromosomes 7, 9, and 10, with six genes on each of these three chromosomes (a cluster of 4 genes on chromosome 7 and a cluster of 6 genes on both chromosomes 9 and 10), followed by chromosome 1 with 5 genes, chromosomes 2 and 16 each with 3 genes, chromosomes 3 and 11 each with 2 genes, and chromosomes 5, 8, 14, and 17 each with one gene. These results indicated that the *crP450* genes were dispersed unevenly across these chromosomes. The genome duplication events of these genes were analyzed, revealing a total of 4 tandem duplications, which were characterized by the presence of numerous members of a gene family in the same intergenic region or in adjacent intergenic regions. Among the four pairs of tandemly duplicated genes identified, two pairs were distributed on chromosome 7, and one pair was distributed on each of chromosomes 9 and 10.

### 3.4. Distribution of Cis-Acting Elements in the Promoter Regions of crP450 Genes in Chlamydomonas reinhardtii

**Figure 4:**
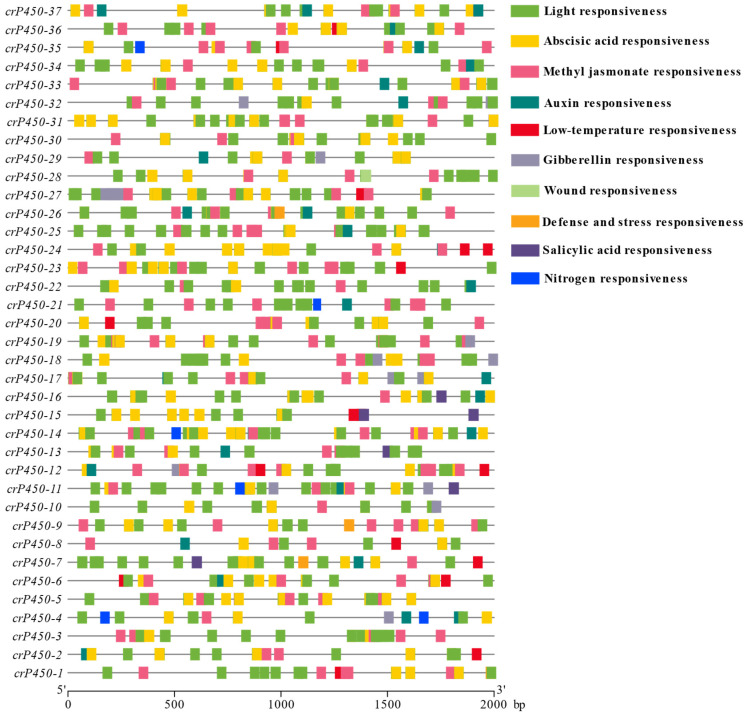
Distribution of *cis*-acting elements in *crP450* genes of *Chlamydomonas reinhardtii*. The ruler represents the length of nucleotide (base pairs).

The results of *cis*-acting element analysis of the 2 kb upstream regions of *crP450* genes revealed the presence of light-, low-temperature-, wound-, defense/stress-, and phytohormone-responsive elements (e.g., abscisic acid-, jasmonic acid-, growth hormone-, gibberellin-, nitrogen-, and salicylic acid-responsive elements) in the *CYP450* gene family (Figure 4). It was noted that both light-responsive elements and two types of phytohormone-responsive elements (i.e., abscisic acid-responsive elements and jasmonic acid-responsive elements) were distributed in all 37 *crP450* genes. Promoter analysis revealed the presence of nitrogen-responsive GATA-box motifs, which were specifically distributed in genes such as *crP450-4* and *crP450-11*. These elements supported the sophisticated regulatory interplay between nitrogen assimilation and the *crP450* family under nutrient stress.

### 3.5. Functional Annotation of crP450 Proteins in Chlamydomonas reinhardtii

**Figure 5:**
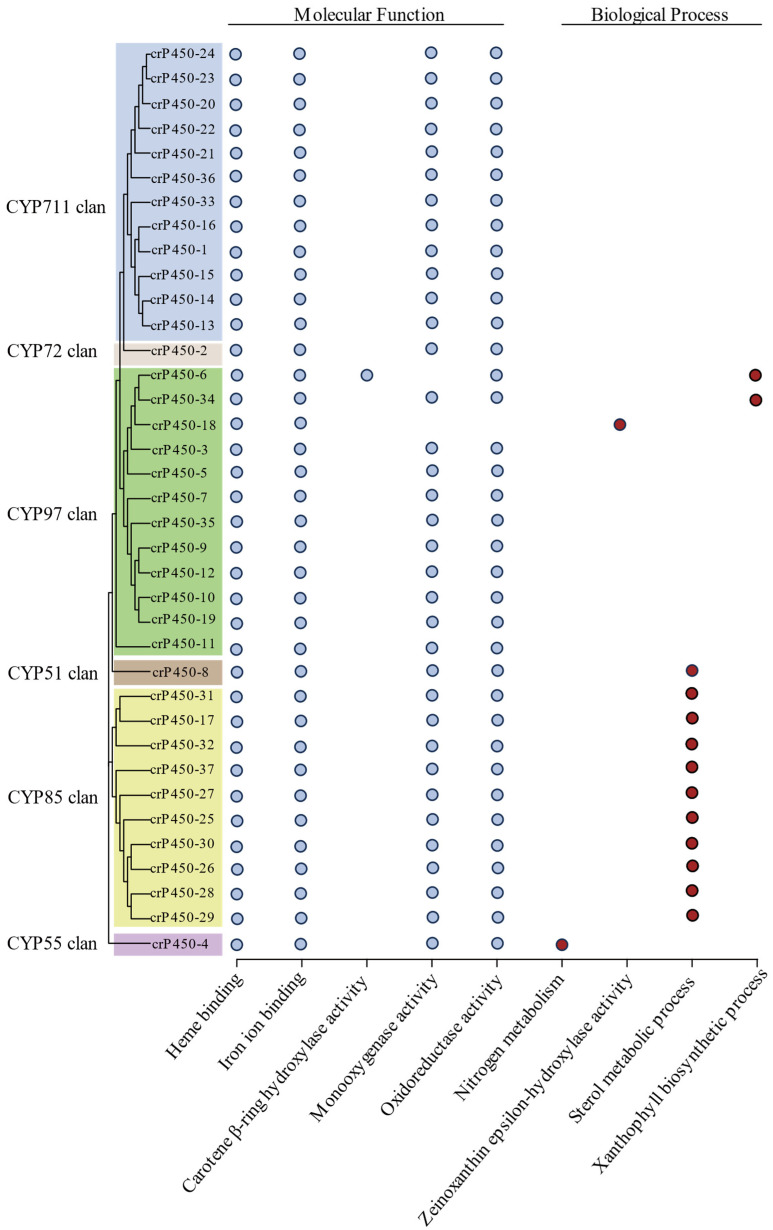
Functional annotation of crP450 enzymes in *Chlamydomonas reinhardtii*. Molecular functions and biological processes with the involvement of crP450 enzymes are indicated in blue and red circles, respectively.

The functional annotation analysis of 37 crP450 proteins was performed using both KEGG and Uniprot databases to determine the molecular functions of crP450 enzymes (Figure 5). The molecular functions of heme binding, iron ion binding, monooxygenase activity, and oxidoreductase activity were commonly detected in all or most crP450 enzymes. The crP450-6 of CYP97 clan was involved in both carotene β-ring hydroxylase activity and xanthophyll biosynthetic process, crP450-34 was involved in xanthophyll biosynthetic process, and crP450-18 was involved in zeinoxanthin epsilon-hydroxylase activity; crP450-8 of CYP51 clan and crP450-18, -31, -17, -32, -37, -27, -25, -30, -26, -28, and -29 of CYP85 clan were involved in sterol metabolic process. Lastly, crP450-4 of CYP55 clan was involved in nitrogen metabolism.

### 3.6. Expression of crP450 Genes in Chlamydomonas reinhardtii Under Different Environmental Conditions

**Figure 6:**
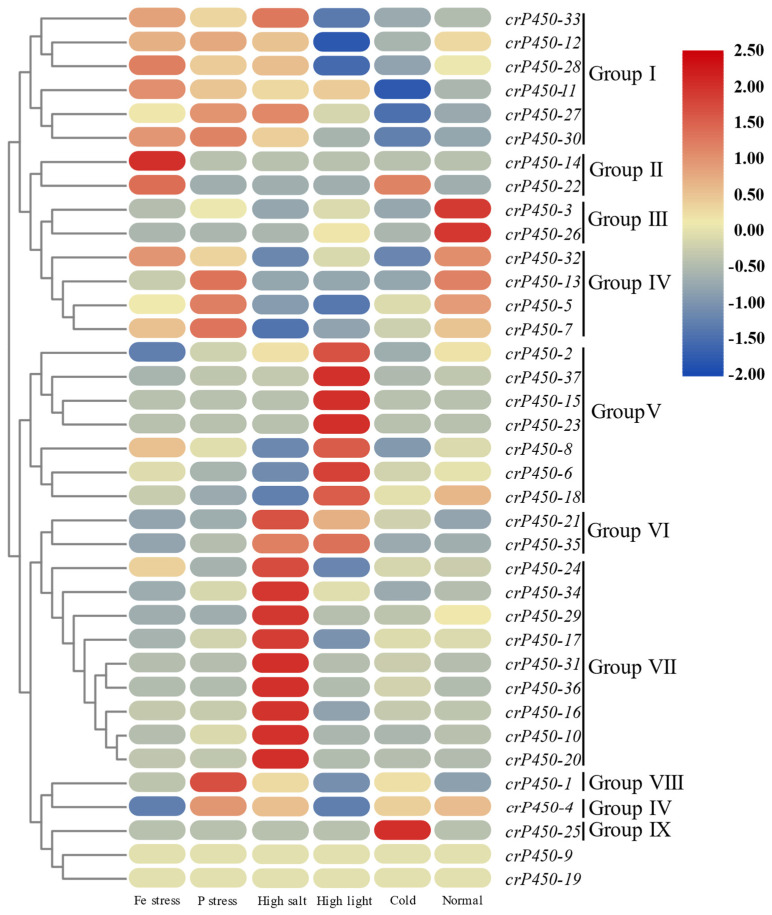
Expression levels of *crP450* genes in *Chlamydomonas reinhardtii* under six different environmental conditions, including iron-deficient (Fe stress), phosphorus-deficient (P stress), high salt, high light, low temperature (cold), and normal environments. High and low gene expression levels are represented in red and blue, respectively.

To evaluate the expression profiles of 37 *crP450* genes across diverse environments, the transcriptome data of *C. reinhardtii* were retrieved from the NCBI SRA database to evaluate their expression patterns (Figure 6). The results revealed these genes in nine groups. Group I contained a total of 6 genes (*crP450-33*, *-12*, *-28*, *-11*, *-27*, and *-30*), showing high expression in iron-deficient environment, phosphorus-deficient environment, and high-salt environment. Group II was composed of two genes, *crP450-14* and *crP450-22*, showing high expression in iron-deficient environment. Two genes were categorized in Group III (*crP450-3* and *crP450-26*), showing high expression in normal environment. Group IV contained a total of five genes (*crP450-32*, *-13*, *-5*, *-7*, and *-4*) with high expression in normal environment and phosphorus-deficient environment. A total of seven genes were revealed in Group V (*crP450-2*, *-37*, *-15*, *-23*, *-8*, *-6*, and *-18*), showing high expression in high-light environment. Two genes were revealed in Group VI (*crP450-21* and *crP450-35*), showing high expression in high-light and high-salt environments. Group VII was composed of a total of nine genes (*crP450-24*, *-34*, *-29*, *-17*, *-31*, *-36*, *-16*, *-10*, and *-20*) with high expression in high-salt environment. Group VIII (*crP450-1*) and Group IX (*crP450-25*) each contained one gene with high expression in phosphorus-deficient environment and low-temperature environment, respectively. Two genes (*crP450-9* and *crP450-19*) showed FPKM values of 0 in all six environments, suggesting that these two genes were either pseudogenes or not expressed under these six environmental conditions.

### 3.7. Quantitative Real-Time PCR Analysis of crP450 Genes in Chlamydomonas reinhardtii

**Figure 7:**
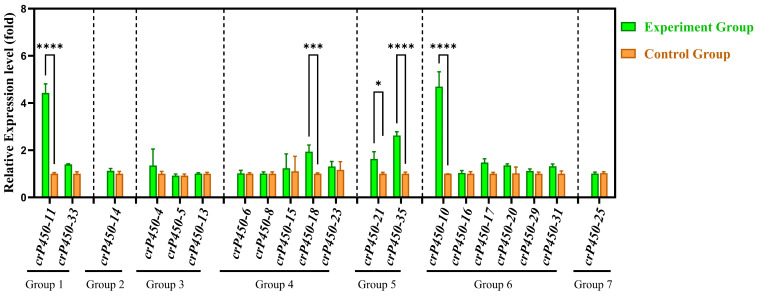
Relative expression of 20 *crP450* genes in *Chlamydomonas reinhardtii* under different treatment conditions based on qRT-PCR analysis of three biological replicates. Genes are categorized into seven groups based on the primary stress conditions that induce significant upregulation patterns. Note that genes in Group 5 exhibit dual responses to high-light and high-salt stresses, whereas genes in Group 6 (including *crP450-10*) are specifically responsive to high-salt stress alone. Group 1 (combined iron, phosphorus, and high-salt stress), Group 2 (iron deficiency), Group 3 (phosphorus deficiency), Group 4 (high light), Group 5 (combined high-light and high-salt stress), Group 6 (high salt), and Group 7 (low temperature). Green bars represent the relative gene expression levels in the experimental group under the specific stress conditions, compared to the control group under normal culture conditions. Error bars indicate standard deviations. Significant differences are determined using Student’s *t*-test based on *p* < 0.05 (*), *p* < 0.001 (***), and *p* < 0.0001 (****), respectively.

To further explore the roles of *crP450* genes, a total of 20 *crP450* genes under six different conditions were randomly selected for qRT-PCR validation to represent diverse phylogenetic clans (covering all major functional groups), varying subcellular localizations, and distinct RNA-seq expression patterns (upregulated, downregulated, and constitutive) (Figure 7). The results revealed a total of seven expression patterns based on their specific stress responses. The gene expression of *crP450-11* was markedly upregulated under iron-deficient, phosphorus-deficient, and high-salt conditions, *crP450-18* was significantly elevated under high-light conditions, *crP450-21* and *crP450-35* were significantly increased under high-light and high-salt conditions, and *crP450-10* was significantly increased under high-salt conditions. No significant differences were detected in the expression of the remaining 17 genes between experimental and control groups. Meanwhile, to quantify the consistency between the two expression profiling platforms, a correlation analysis was performed between the log2(Fold Change) values derived from RNA-seq and qRT-PCR. The results showed a significant positive correlation (Pearson’s r = 0.55, *p* < 0.05), confirming that the directional expression trends of the 20 sampled crP450 genes were consistent between both methods (Figure S3).

## 4. Discussion

CYP450 enzymes are generally involved in the synthesis of membrane sterols, phytohormones, and signaling molecules in many prokaryotic and eukaryotic organisms, whereas CYP450s in humans and other higher animals mediate the degradation of drugs, toxins, and other foreign compounds [50]. With the rapid advancement in next-generation sequencing technology, numerous *CYP450* gene families have been identified [51,52,53]. However, the understanding of the functions of algal *CYP450s* is still limited [54]. In this study, we identified and further characterized the *CYP450* gene family members of *C. reinhardtii*. This investigation established a strong theoretical basis for further exploration of the functions of *CYP450s* in *C. reinhardtii*.

The results of genome-wide analysis of the *CYP450* gene family of *C. reinhardtii* revealed a total of 37 *CYP450* genes, and the CYP450 enzymes were further characterized to evaluate their pI values, molecular masses, subcellular localizations, and signal peptides. The results showed that a larger number of *CYP450* genes was detected in *C. reinhardtii* than that in cyanobacteria [55], which was probably attributed to the larger genome size of *C. reinhardtii* compared to cyanobacteria. However, the number of *CYP450* genes in *C. reinhardtii* was much less than those in plants; e.g., a total of 95, 326, 273, and 384 *CYP450* genes have been identified in sweet potato, rice, tea, and oilseed rape, respectively [53,56,57,58]. The large quantity and functional versatility of CYP450s are likely due to the larger plant genomes compared to microalgae and the more complex structure and function of plant proteins. Similarly, a total of 122 *CYP450* genes in 11 gene families are revealed in the fungal genome of *Conidiobolus heterosporus* [59]. Although varied numbers of *CYP450* genes are reported in algal taxa, ranging from 19 in *Volvox carteri* to 1199 in *Prasinoderma coloniale*, the number of *CYP450* genes in *C. reinhardtii* was comparable with most algal taxa, e.g., 58 in *Trentepohlia annulata* and 46 in *Penium margaritaceum* [33].

The gene structure, chromosomal localization, and gene duplication of the *crP450* genes of *C. reinhardtii* were further analyzed using bioinformatics methods. The results demonstrated that the 37 *crP450* genes were localized to 12 chromosomes and the CYP450 enzymes were localized in various organelles, e.g., 20 in chloroplast, followed by 5 in cytoplasm, 4 in both mitochondria and cytoplasmic membrane, 2 in endoplasmic reticulum, and one in both extracellular and plasma membranes. These results aligned with those previously reported, e.g., the *CYP450* genes of tea were mainly localized in chloroplast, plasma membrane, and cytoplasm [57], and the *CYP450* genes of *Brassica napus* and *Fritillaria cirrhosa* were largely localized in chloroplast [58]. The distribution of CYPs is inextricably linked to their functions and substrates. For example, heterologous expression of the *CYP450s* is frequently technically challenging and requires membrane-bound NADPH-dependent reductase, and heterologous *CYP450s* are expressed in the chloroplasts of *Nicotiana benthamiana*, replacing the natural reductase with a photosynthetic apparatus function through the endogenous soluble electron carrier ferric oxidoreductase [60]. CYP74A (propadiene oxide synthase) localized in chloroplasts is involved in the biosynthesis of jasmonic acid, whereas another CYP450 hydrogen peroxide cleavage enzyme (HPL; CYP74B) is also located in chloroplasts, competing with CYP74A for a common substrate [61]. The functions of all these CYP450s localized in chloroplasts are mainly involved in the metabolic pathways in chloroplasts. This is consistent with the chloroplast being the primary site for fatty acid biosynthesis and starch accumulation in *C. reinhardtii*, suggesting that these CYP450s may regulate carbon flux toward these energy storage compounds. Previous studies showed that a total of 116 *CYP450* genes in *Setaria italica* were distributed in 33 tandemly duplicated gene clusters on 7 chromosomes, and a total of 20 conserved motifs were identified [62]. In our study, the analysis of genome duplication events in *C. reinhardtii CYP450* genes revealed four pairs of tandemly duplicated genes. It is well known that tandem gene duplications contribute significantly to the composition of the *CYP450* gene family. For example, due to the tandem duplication of genes during evolution, *CYP450* genes are frequently amplified, resulting in new alleles with new or enhanced functions of the *CYP450* genes [63]. In our study, clustering analysis of gene expression patterns of 37 *crP450* genes revealed varied expression patterns among several pairs of tandemly duplicated genes in *C. reinhardtii* under different environmental conditions, suggesting that these genes played different roles under different environments, while variations in gene expression played an important role in the conservation of duplicated genes.

CYP450s usually contribute to regulating plant responses to environmental stresses and phytohormone pathways [64]. The *CYP450* genes encode enzymes that are involved in a variety of biochemical reactions and are the largest protein family in plants. In our study, light-, low-temperature-, wound-, defense-, stress-, and phytohormone-responsive elements were detected in the promoter regions of *crP450* genes of *C. reinhardtii*, with both light-responsive elements and two phytohormone-responsive elements (abscisic acid-responsive elements and jasmonic acid-responsive elements) shared among all *crP450* genes. These findings were consistent with the results published previously. For example, the promoter regions of *CYP450* genes in *Brassica napus* and *Camellia sinensis* contain a variety of *cis*-acting elements related to phytohormone and adversity response [57,58], and an abundance of *cis*-acting elements associated with light response, hormone-regulated, and stress-related signaling pathways is also detected in the promoter regions of the *CYP71* genes of *Oryza sativa* subsp. *indica* [65]. Furthermore, the promoter regions of all *CYP450* genes in *Setaria italica* contain the hormone-responsive elements [62], and the *CYP450* genes identified in *Pyrus bretschneideri* contain hormone-responsive, light-responsive, hypoxia-specific inducible, and anaerobic-inducible *cis*-acting elements [66]. These studies are consistent with the *cis*-acting elements revealed in the present study, suggesting the shared *CYP450* regulatory mechanisms in plants and microalgae, which is crucial to improve the understanding of *CYP450* regulation in microalgae. For example, previous studies showed that *OsCYP71D8L*, a *CYP450* member of the CYP71 clan in *Oryza sativa*, was involved in the regulation of multiple agronomic traits and abiotic stress responses through influencing gibberellin and cytokinin homeostasis levels [67], and three differentially expressed *CYP450* genes were detected in *Flammulina velutipes* under green and blue light treatments [68]. The specific expression patterns revealed in this study indicate that *C. reinhardtii* dynamically reprograms its CYP450 metabolic network to respond to environmental stress damage. For instance, under high-light stress, the significant upregulation of Group V genes (*crP450-6* and *crP450-18*) aligns with their predicted roles in lutein biosynthesis. Increased expression of these hydroxylases may promote accumulation of photoprotective pigments like zeaxanthin, thereby scavenging reactive oxygen species and preventing photooxidative damage. Similarly, the upregulation of sterol biosynthesis genes (CYP51 and CYP85 families) under salt stress suggests a mechanism to counteract osmotic stress damage by enhancing membrane stability and fluidity. These transcriptional changes indicate that *crP450* genes are not merely passive markers of stress responses but active drivers of metabolic adaptive regulation. Further studies are necessary to evaluate the functions of these *crP450* genes in *C. reinhardtii* [69].

Understanding the functions of genes helps improve genetic engineering of the target species for desired traits. In our study, the varied expression levels of a group of selected *crP450* genes in *C. reinhardtii* under different environments were detected using qRT-PCR. In *Arabidopsis thaliana*, the upstream regulator WRKY9 controls the corky substance deposition by regulating two CYP450 enzymes (CYP94B3 and CYP86B1), ultimately enhancing plant salt tolerance [70], and CYP714D1 of rice is overexpressed in poplar to improve gibberellin content and plant salt tolerance [71]. Here, we further discuss the functions of *crP450 genes* of different CYP450 clans in *C. reinhardtii*.

In the CYP711 clan, we identified five *C. reinhardtii* members (*crP450-13*, *-14*, *-21*, *-22*, and *-36*). Our *cis*-element analysis revealed that these genes are enriched with growth hormone-responsive elements (Figure 4). In land plants like *Arabidopsis*, the CYP711 homolog MAX1 regulates axillary meristem formation via strigolactone signaling. Although *C. reinhardtii* is unicellular and lacks meristems, the conservation of this clan and the presence of hormone-responsive promoters suggest that these *crP450s* may regulate ancient signaling pathways governing cell density or population growth. This hypothesis is supported by the specific upregulation of *crP450-21* under combined high-light and high-salt stress (Group 5 in Figure 7), indicating a potential role in modulating cell cycle progression under environmental stress.

Members of the CYP51 (*crP450-8*) and CYP85 clans (*crP450-17*, *-25 to -32*, and *-37*) in *C. reinhardtii* appear to play a pivotal role in maintaining membrane integrity. Our RNA-seq data placed several of these genes (*crP450-28*, *-27*, and *-30*) into Group I, characterized by high expression under high-salt and nutrient-deficient conditions (Figure 6). Similarly, qRT-PCR confirmed that *crP450-10* (CYP51 clan) was significantly upregulated under salt stress (Figure 7). In other organisms, CYP51 and CYP85 catalyzing are the key steps in sterol biosynthesis [72]. Therefore, we propose that the upregulation of these genes in *C. reinhardtii* enhances sterol production to reinforce the cell membrane against osmotic stress, a mechanism analogous to the salt-induced sterol modulation observed in halotolerant yeast and higher plants.

CYP51 is an enzyme required for the synthesis of sterols in eukaryotes. It is a main target of antifungal drugs and a prospective target for the treatment of protozoan infections [73]. Goldstone et al. identified recombinant CYP450 sterol 14α-demethylase (a form of CYP51) from the deep-sea fish, *Coryphaenoides armatus*, showing that heterologous expression of CYP51 in *Escherichia coli* catalyzed the 14α-demethylation of lanosterol [74]. The *CYP51* gene of *Xanthophyllomyces dendrorhous* encodes a functional sterol C14 demethylase, which is involved in ergosterol biosynthesis [75]. Heterologous expression of CYP51 from *Dioscorea transversa* in *E. coli* can demethylate obtusifoliol, which is an intermediate product in phytosterol biosynthesis, and possibly 4-desmethyl-24,25-dihydrolanosterol, a downstream intermediate in cholesterol biosynthesis [72]. A CYP450 detected in *Methylobacterium anomalum* with strong homology to CYP51 was expressed in *E. coli* to generate CYP450 with a molecular weight of about 62 kDa and bound to lanosterol as a putative substrate, exerting sterol 14-demethylase activity based on gas chromatography/mass spectrometry [76]. These results were consistent with our study, suggesting that *crP450-8* in the CYP51 clan is putatively involved in the steroid biosynthesis pathway.

CYP450 enzymes of the CYP85 clan catalyze oxidative reactions, which are essential for the biosynthesis of the oleuropein steroid hormone [77]. In our study, a group of genes in CYP85 clan (i.e., *crP450-17*, *crP450-25* to *crP450-32*, and *crP450-37*) are predicted to participate in the metabolic process of sterols, which are a class of steroids. These results were consistent with those previously reported. For example, *Arabidopsis* CYP85 is involved in steroid metabolism; CYP85A1 catalyzes the oxidation of the 6-deoxy intermediate C-6, and CYP85A2 is involved in oleuropein steroid synthesis [78]. Furthermore, heterologous expression of *Lycopersicon esculentum*-derived and *Arabidopsis*-derived CYP85 in yeast revealed that both CYP85s catalyze multiple steps in steroid biosynthesis, including the generation of thaumatinone from 6-deoxytheasterone, 3-dehydro-6-deoxytheasterone from 3-dehydrotheasterone, 6-deoxychromanosterol from chromanosterol, and 6-deoxyricinosterone from ricinosterone [79].

As a model system for the nitrogen cycle in microalgae, *C. reinhardtii* possesses a complete pathway for nitrate assimilation and nitric oxide (NO) reduction. Previous studies have documented that this species actively synthesizes nitrous oxide (N_2_O) under dark anoxic conditions [60]. Our study identifies *crP450-4* (CYP55) as the putative nitric oxide reductase (P450nor) responsible for this critical step in the algal nitrogen cycle, linking the CYP450 family to greenhouse gas metabolism. The nitrate/nitrite-inducible CYP450 of *Fusarium spinosum*, isolated by immunoscreening methods, is identified as CYP55, which is involved in the heterogeneous reduction of nitrite by the fungus [80]. Overexpression of CYP55A5 in *Aspergillus oryzae* resulted in the intracellular accumulation of a large quantity of active proteins, which utilized both NADH and NADPH as electron donors for the reduction of nitric oxide to nitrous oxide [81]. These findings are consistent with our study, showing that *crP450-4* in the CYP55 clan encoded a fungal nitric oxide reductase involved in nitrogen metabolism, and the low expression of *crP450-4* under high-light conditions (Figure 5) was consistent with the function of CYP55 in N_2_O synthesis under dark conditions [19]. Our transcriptome analysis placed *crP450-4* in Group IV, characterized by sustained high expression under both normal and phosphorus-deficient conditions (Figure 6). While *crP450-4* is a predicted P450nor involved in nitrogen metabolism, its activity during phosphorus starvation highlights the critical nitrogen-phosphorus nutrient crosstalk in microalgae. Severe phosphorus limitation in plants can disrupt normal cellular metabolism and lead to imbalances in nitrogen metabolism, including increases in reactive nitrogen species such as nitric oxide (NO), which are part of broader stress responses involving reactive oxygen and nitrogen signaling [82,83]. Therefore, we propose that the constitutive expression of *crP450-4* under phosphorus deficiency serves a detoxifying role, converting excess intracellular NO into N_2_O to prevent nitrosative stress, a mechanism essential for survival even when external nitrogen is abundant. Future studies are necessary to incorporate nitrogen deficiency treatments to fully elucidate its regulatory characteristics during gametogenesis and nitrogen starvation processes.

The CYP97 clan members (*crP450-6*, *-18*, *-34*, and *-35*) exhibited expression patterns strongly indicative of a photoprotective function. For example, *crP450-18*, predicted to possess zeinoxanthin epsilon-hydroxylase activity, was identified in Group V (RNA-seq), showing significant upregulation under high-light treatment in qRT-PCR analysis (Figure 7). In plants like *Arabidopsis*, CYP97 enzymes are essential for hydroxylation steps in the carotenoid pathway that produce lutein [84]. The specific induction of *crP450-18* and *crP450-6* by high light suggests that *C. reinhardtii* actively upregulates these hydroxylases to increase xanthophyll accumulation, thereby dissipating excess light energy and preventing photooxidative damage.

The CYP97 clan contains enzymes involved in carotenoid synthesis, which is crucial for photosynthesis and photoprotection. Previous studies showed that heterologous expression of PrtCYP97B2 derived from *Phaeodactylum tricornutum* in *E. coli* revealed that PrtCYP97B2 catalyzed the hydroxylation of the β-carotene β-ring, leading to the biosynthesis of zeaxanthin in β-carotene-accumulating *E. coli* BL21 (DE3) cells [85]. In carrots, CYP97A3 catalyzes the hydroxylation of α-carotene to produce zeaxanthin, which is then hydroxylated by CYP97C1 to produce lutein [86]. In *Oriza sativa*, CYP97A4 functions as a β-carotene dihydroxylase [87]. In *Nelumbo nucifera*, CYP97H1 catalyzes the monohydroxylation of β-carotene to produce β-cryptoxanthin [88]. In our study, *crP450-6* was predicted to be involved in xanthophyll biosynthetic process, showing carotene β-ring hydroxylase activity, whereas *crP45-18* likely exhibited zeinoxanthin epsilon-hydroxylase activity, and was highly expressed under high-light conditions. It is commonly known that both high-light and high-salt conditions are stresses to induce zeinoxanthin synthesis in microalgae [89]; the synthesis of carotenoid pigments is enhanced, and the expression of genes involved in the synthesis of carotenoid pigments is increased to enhance the biosynthesis of carotenoids, which act as photoprotective agents. In our study, the *crP450-35* gene was highly expressed under high-light and high-salt conditions, and the *crP450-10* gene was highly expressed under high-salt conditions, suggesting that both *crP450-35* and *crP450-10* genes are putative candidates that were probably involved in the synthesis of carotenoids.

CYP72 is primarily implicated in the metabolism of diverse hydrophobic compounds, such as fatty acids, isoprenoids, and hormones [90] (e.g., oleoresin steroids and gibberellins), and the biosynthesis of cytokinins. However, the functions of crP450-2 in the CYP72 clan were not revealed in our study. Further studies are necessary to explore the functions of this gene in *C. reinhardtii*.

Because cytochrome P450 enzymes are iron-dependent heme-thiolate proteins, significant changes in their expression can reflect broader shifts in cellular iron allocation and homeostasis. In *Chlamydomonas reinhardtii*, iron deficiency triggers an iron-sparing response in which non-essential iron-rich proteins, including certain ferredoxin isoforms, are downregulated, helping conserve iron for critical functions such as photosynthesis and stress responses [91]. Interestingly, our results revealed robust upregulation of Group I and Group II genes (e.g., *crP450-11* and *crP450-14*) under iron stress (Figure 6). This paradox implies that the catalytic products of these specific enzymes—likely sterols or stress-signaling molecules—are indispensable for survival, necessitating the allocation of scarce iron to their holoenzymes despite the metabolic cost.

Additionally, functional links exist between the CYP450 family and other metalloenzymes [19]. Specifically, *crP450-4* (a CYP55/P450nor homolog) functions downstream of nitrate reductase, a molybdenum (Mo)-dependent enzyme. The coordinated activity of the Mo-dependent nitrate reductase (producing nitrite/NO) and the heme-dependent P450nor (detoxifying NO) underscores the complex interplay between Iron and molybdenum networks in regulating nitrogen assimilation and preventing cytotoxicity.

## 5. Conclusions

In this study, we conducted a comprehensive genome-wide identification and characterization of the *CYP450* gene family in the model alga *C. reinhardtii*. A total of 37 *crP450* genes were identified and classified into six distinct clans (CYP711, CYP72, CYP97, CYP51, CYP85, and CYP55). Subcellular localization analysis predicted that the majority of these enzymes (20 members) are located in the chloroplasts, suggesting their potential roles in photosynthesis-related metabolic pathways.

Promoter analysis revealed a variety of *cis*-acting elements responsive to light, phytohormones, and abiotic stresses. This was corroborated by transcriptomic and qRT-PCR analyses, which identified distinct gene clusters responding to specific environmental challenges. Notably, Group I genes (*crP450-11*) were strongly upregulated under nutrient deficiency (iron/phosphorus) and salt stress, while Group V genes (*crP450-18*) were significantly induced under high-light conditions, potentially participating in photoprotective mechanisms such as carotenoid biosynthesis.

Overall, these findings provide a solid theoretical foundation for understanding the regulatory mechanisms of algal CYP450s. The identification of stress-responsive candidate genes such as *crP450-11* and *crP450-18* offers putative targets for future genetic engineering strategies aimed at enhancing stress resilience in industrial microalgae.

## Figures and Tables

**Table 1:** Molecular Characteristics of *crP450* Genes of *Chlamydomonas reinhardtii*.

Name	Gene ID	Intron	Chromosomal Location	Amino Acid	Molecular Weight (Da)	Isoelectric Point	Subcellular Localization
*crP450-1*	CHLRE_01g054250v5	14	1: 7,527,783–7,534,487	706	73,840.9	8.7	Chloroplast
*crP450-2*	CHLRE_01g038500v5	6	1: 5,466,990–5,4715,05	751	77,097.1	7.0	Chloroplast
*crP450-3*	CHLRE_01g027550v5	8	1: 4,167,300–4,171,523	381	41,742.8	5.6	Cytoplasm
*crP450-4*	CHLRE_01g007950v5	10	1: 1,533,342–1,539,057	400	43,851.2	6.4	Mitochondria
*crP450-5*	CHLRE_01g003850v5	13	1: 739,299–746,905	523	57,140.1	6.1	Cytoplasm
*crP450-6*	CHLRE_02g142266v5	15	2: 8,719,899–8,725,739	652	71,728.5	6.9	Mitochondria
*crP450-7*	CHLRE_02g144250v5	9	2: 7,452,001–7,457,652	675	70,849.0	8.5	Plasma membrane
*crP450-8*	CHLRE_02g092350v5	9	2: 2,518,335–2,523,186	495	56,044.7	7.7	Chloroplast
*crP450-9*	CHLRE_03g166900v5	12	3: 3,401,931–3,410,088	1029	107,301.3	9.3	Plasma membrane
*crP450-10*	CHLRE_03g161250v5	10	3: 2,687,264–2,694,137	882	88,811.2	8.0	Nuclear envelope
*crP450-11*	CHLRE_05g234100v5	8	5: 204,046–209,162	543	60,041.7	7.7	Endoplasmic reticulum
*crP450-12*	CHLRE_07g356250v5	14	7: 6,181,676–6,189,645	532	58,689.1	9.6	Chloroplast
*crP450-13*	CHLRE_07g354450v5	8	7: 5,950,163–5,955,057	491	51,529.4	7.2	Mitochondria
*crP450-14*	CHLRE_07g354400v5	13	7: 5,945,283–5,950,037	628	66,974.8	9.2	Chloroplast
*crP450-15*	CHLRE_07g354350v5	10	7: 5,940,143–5,945,234	551	57,995.7	9.0	Chloroplast
*crP450-16*	CHLRE_07g340850v5	15	7: 4,414,119–4,420,514	643	68,232.1	8.8	Chloroplast
*crP450-17*	CHLRE_07g325000v5	8	7: 1,608,757–1,612,723	564	58,573.3	9.1	Chloroplast
*crP450-18*	CHLRE_08g373100v5	16	8: 2,653,596–2,661,147	586	63,466.3	6.8	Mitochondria
*crP450-19*	CHLRE_09g399402v5	19	9: 5,215,453–5,223,494	618	66,500.6	8.7	Extracellular
*crP450-20*	CHLRE_09g397734v5	15	9: 4,872,845–4,880,100	611	64,464.9	8.6	Chloroplast
*crP450-21*	CHLRE_09g397216v5	15	9: 4,749,601–4,757,454	605	64,893.5	8.0	Chloroplast
*crP450-22*	CHLRE_09g397105v5	15	9: 4,717,525–4,724,110	568	60,884.3	8.4	Chloroplast
*crP450-23*	CHLRE_09g397031v5	12	9: 4,708,111–4,714,302	544	57,930.2	8.5	Chloroplast
*crP450-24*	CHLRE_09g396994v5	14	9: 4,702,791–4,7080,62	607	65,023.1	8.5	Endoplasmic reticulum
*crP450-25*	CHLRE_10g427500v5	8	10: 1,308,342–1,312,377	483	51,879.0	8.3	Cytoplasm
*crP450-26*	CHLRE_10g427350v5	12	10: 1,290,792–1,296,247	484	53,995.7	7.2	Mitochondria
*crP450-27*	CHLRE_10g426950v5	9	10: 1,267,438–1,271,690	507	55,200.6	6.4	Chloroplast
*crP450-28*	CHLRE_10g426750v5	11	10: 1,246,442–1,252,817	515	56,349.8	8.6	Chloroplast
*crP450-29*	CHLRE_10g426700v5	10	10: 1,240,844–1,246,377	547	59,979.7	7.3	Chloroplast
*crP450-30*	CHLRE_10g426600v5	12	10: 1,227,452–1,233,617	518	57,271.5	7.2	Chloroplast
*crP450-31*	CHLRE_11g467627v5	14	11: 706,557–712,788	561	58,649.2	8.2	Plasma membrane
*crP450-32*	CHLRE_11g467527v5	9	11: 40,146–45,687	515	57,623.2	7.7	Cytoplasm
*crP450-33*	CHLRE_14g626400v5	15	14: 2,651,670–2,659,603	673	69,676.2	9.3	Chloroplast
*crP450-34*	CHLRE_16g678437v5	14	16: 7,029,700–7,035,865	650	67,523.8	8.3	Chloroplast
*crP450-35*	CHLRE_16g659200v5	11	16: 2,300,238–2,306,468	662	69,629.3	6.6	Chloroplast
*crP450-36*	CHLRE_16g648200v5	14	16: 886,828–893,823	633	66,964.6	8.9	Chloroplast
*crP450-37*	CHLRE_17g731750v5	11	17: 4,415,984–4,424,159	600	64,537.8	9.1	Plasma membrane

**Table 2:** Motifs detected in crP450 proteins of *Chlamydomonas reinhardtii*.

Name	Sequence (# of Amino Acids)
motif-1	AYLPFGGGPRMCVGQKLAMMEAKVALALLLRRYRFELHPPQ (41)
motif-2	DLPRLPYLEAVVKEALRLYPP (21)
motif-3	YSLHRDPAVWPRPEAFRPERF (21)
motif-4	WYHAYLMHCJDPVLWDGDTSVDVPAHMDWRNNFEGAFRPERWLSEETKPK (50)
motif-5	MLFPELRPLLRWLAHHLPDAAQTRHMRARTKLANVSRQLMESWKAQKAA (49)
motif-6	FLLAGYETTAAALAW (15)
motif-7	YLLATHPEVQARLLAEVDAVL (21)
motif-8	GRLTLDVVGETAYGVDFGSLE (21)
motif-9	NAGAFVASGEVWRRGRRVFEASIIHPASLAAHLPAINRC (39)
motif-10	WFGVRPWIVIADPALIRKLAYKCLARPASMSEYGHVLTGEN (41)

## Data Availability

Raw data can be provided according to requirements. If needed, please contact the corresponding author.

## Supplementary Materials

The following supporting information can be downloaded at: https://www.mdpi.com/article/10.3390/biology15010077/s1, Figure S1: Sequence Logo of conserved motifs in a total of 37 *Chlamydomonas reinhardtii* CYP450 protein sequences using WebLogo; Figure S2: Morphological features and cultural conditions of *Chlamydomonas reinhardtii* FACHB-265; and Figure S3: Correlation of gene expression levels between RNA-seq and qRT-PCR; Table S1: Composition of TAP medium.
