# Supplementary material for: Genome-Wide Characterization and Expression Analysis of CYP450 Genes in Chlamydomonas reinhardtii P.A. Dang
Source: Biology (Basel). 2025 Dec 31;15(1):77. doi: 10.3390/biology15010077 (PMC12784940; doi:10.3390/biology15010077)
Supplement: Supplementary file 1 [file biology-15-00077-s001.zip › biology-4056359-supplementary.pdf]

Supplementary materials

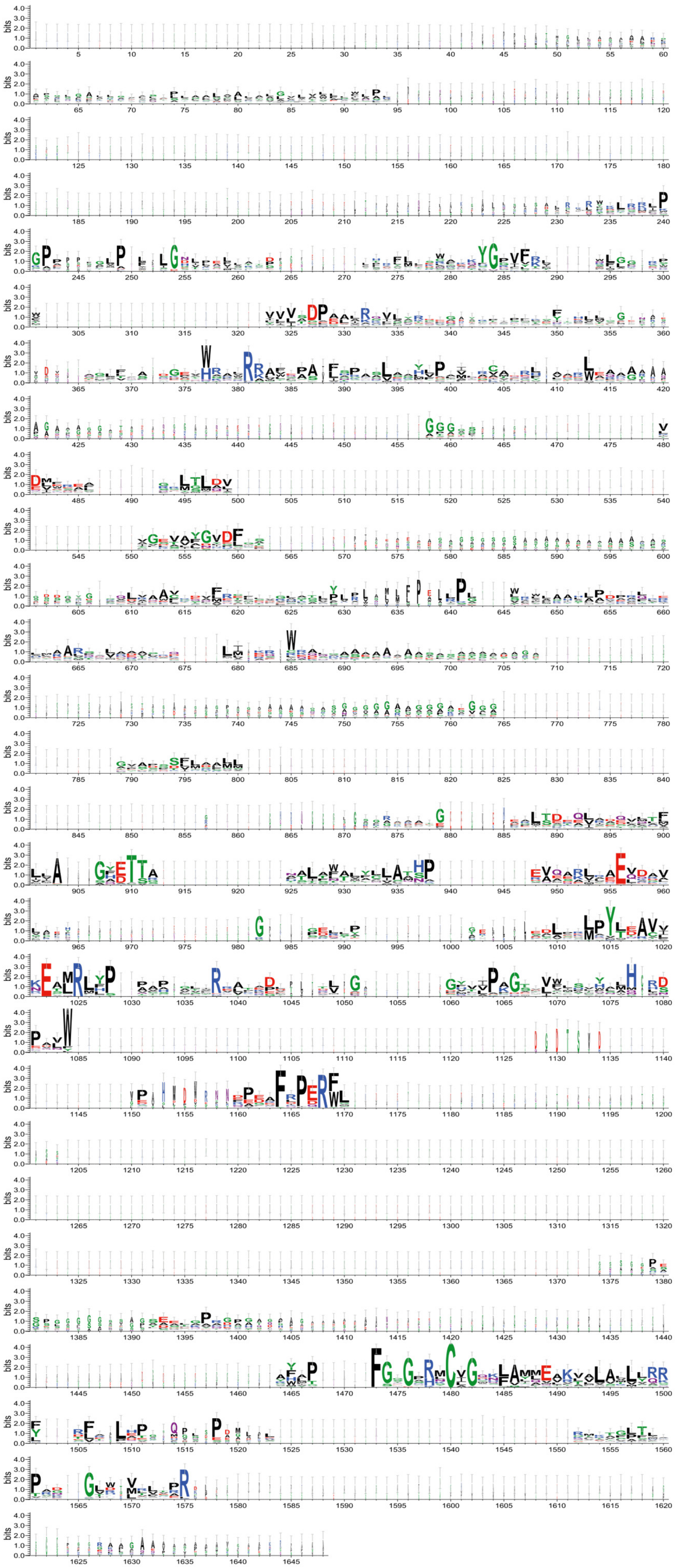

Figure S1. Sequence Logo of conserved motifs in a total of 37 *Chlamydomonas reinhardtii* CYP450 protein sequences based on WebLogo. The logo is derived from the alignment of the 37 *crP450* genes listed in Table 1. The height of each stack indicates the sequence conservation at that position (measured in bits), and the height of amino acid symbols within the stack indicates the relative frequency of each amino acid.

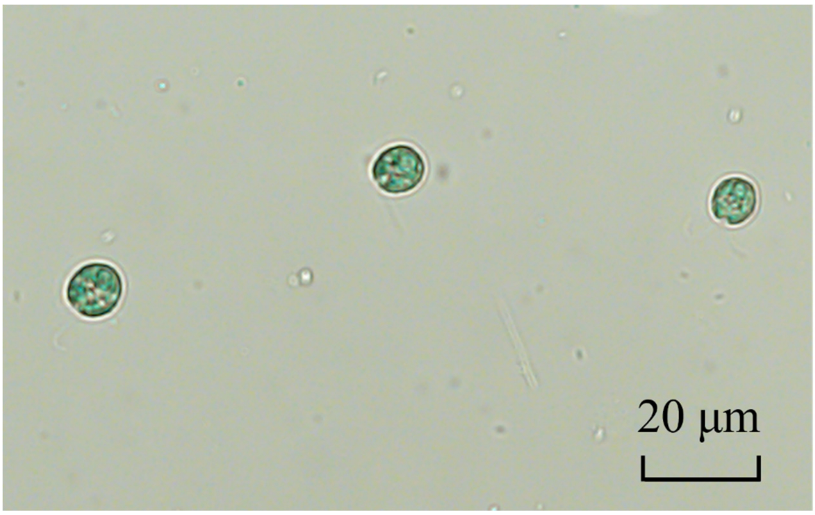

Figure S2. Morphological features and cultural conditions of *Chlamydomonas reinhardtii* FACHB-265.

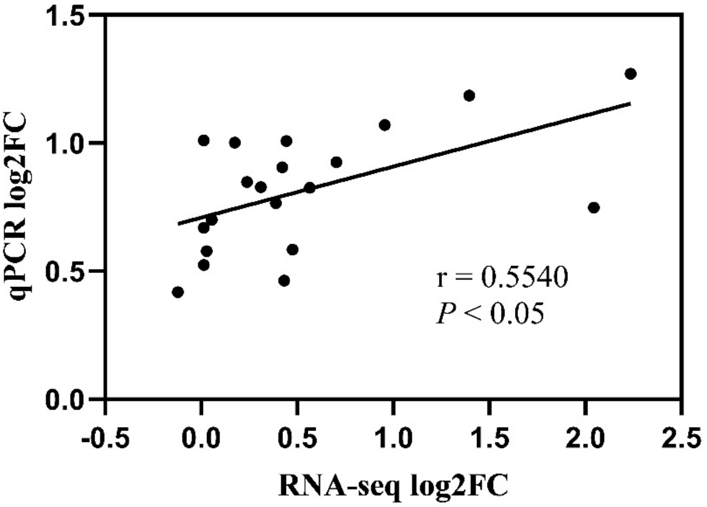

Figure S3. Correlation of gene expression levels between RNA-seq and qRT-PCR. The scatter plot illustrates the correlation between the log_2(Fold Change) values obtained from transcriptome sequencing (X-axis) and qRT-PCR analysis (Y-axis) for 20 selected *crP450* genes. A positive correlation (Pearson's r= 0.55, $P < 0.05$) indicates that the directional trends of gene expression are consistent across both platforms[17]. Discrepancies in absolute magnitude are attributed to the higher sensitivity and different normalization methods (FPKM vs. $2^{-\Delta\Delta Ct}$) of the two techniques.

Table S1. Composition of TAP medium. Iron-deficient TAP medium contains no iron source ($FeSO_4{\cdot}7H_2O$); phosphorus-deficient TAP medium contains no phosphorus sources (K_2HPO_4 and KH_2PO_4).

Component	g/L
Tris base	2.42
NH_4Cl	0.38
$MgSO_4·7H_2O$	0.10
$CaCl_2·2H_2O$	0.057
K_2HPO_4	0.11
KH_2PO_4	0.054
EDTA-2Na	0.05
$ZnSO_4·7H_2O$	0.022
H_3BO_3	0.0114
$MnCl_2·4H_2O$	0.00506
$CoCl_2·6H_2O$	0.00161
$CuSO_4·5H_2O$	0.00157
$(NH_4)_2Mo_7O_{24}·4H_2O$	0.0011
$FeSO_4·7H_2O$	0.00499
CH_4COOH	0.001 mL
